# The Efficacy of Transarterial Chemoembolization plus Apatinib or Sorafenib in the Treatment of Advanced Hepatocellular Carcinoma

**DOI:** 10.1155/2021/8169012

**Published:** 2021-11-19

**Authors:** Lei Chen, Tao Sun, Linxia Wu, Weihua Zhang, Yanqiao Ren, Dongqiao Xiang, Bin Liang, Chuansheng Zheng

**Affiliations:** ^1^Department of Radiology, Union Hospital, Tongji Medical College, Huazhong University of Science and Technology, Wuhan 430022, China; ^2^Hubei Province Key Laboratory of Molecular Imaging, Wuhan 430022, China; ^3^Department of Interventional Radiology, Union Hospital, Tongji Medical College, Huazhong University of Science and Technology, Wuhan 430022, China

## Abstract

**Background:**

Transarterial chemoembolization (TACE) combined with sorafenib (TACE-S) or apatinib (TACE-A) is used in the treatment of hepatocellular carcinoma (HCC). However, to date, no study has compared the efficacy and safety of both treatments. The objective of this study was to compare the efficacy and safety of patients with advanced HCC who received either TACE-S or TACE-A.

**Methods:**

193 patients with advanced HCC were included in the study between June 2015 and December 2019. Propensity score matching (PSM) analysis was used in the study to reduce selection bias.

**Results:**

Before PSM, the median overall survival (mOS) and median progression-free survival (mPFS) of patients treated with TACE-S were not significantly longer than in patients treated with TACE-A (*P*=0.703, *P*=0.514). TACE-A did not increase the mortality risk compared with TACE-S in the first 12 months (HR: 1.255, 95%CI: 0.796–1.978, *P*=0.329) or after the 12-month mark (HR: 0.832, 95%CI: 0.482–1.436, *p*=0.508). Similarly, TACE-A did not increase the tumor recurrence risk relative to TACE-S in the first 12 months (HR: 1.054, 95%CI: 0.744–1.493, *P*=0.767) or after the 12-month mark (HR: 1.730, 95%CI: 0.592–5.049, *P*=0.316). The subgroups analysis showed that TACE-A did not increase mortality risk or tumor recurrence risk relative to TACE-S. After PSM, similar results were presented. The III and IV stage adverse events in the TACE-A group were similar to those in the TACE-S group before PSM.

**Conclusions:**

Patients with advanced hepatocellular carcinoma could get similar survival benefits from treatment with either transarterial chemoembolization plus apatinib or transarterial chemoembolization plus sorafenib.

## 1. Introduction

Hepatocellular carcinoma (HCC) is one of the most common cancers in the world [1]. At present, there are 841,000 new cases of HCC worldwide every year, and 46.71% of the new cases are from China [2]. The age-standardized incidence rate per 100,000 is 26.8 in Eastern Asia [1]. Patients with early HCC (Barcelona clinic liver cancer (BCLC) stage 0 or A) have better survival benefits from radical treatment, such as liver transplantation, liver resection, or radiofrequency ablation. Patients with intermediate HCC are recommended transarterial chemoembolization (TACE) as the first-line treatment, according to the European Association for the Study of the Liver (EASL) because it can extend the two-year survival rate of patients [3, 4]. However, studies have shown that patients with advanced HCC could also get survival benefits from TACE [5–7]. For patients with advanced HCC, the SHARP trial showed that the median overall survival (OS) of patients with advanced HCC who received sorafenib was 2.8 months longer than that of patients who received a placebo [8]. Sorafenib is therefore recommended as the first-line treatment for patients with advanced HCC. However, some patients did not see obvious survival benefits from sorafenib alone due to the low treatment response rate. Thus, sorafenib combined with other treatments can be used in the treatment of patients with advanced HCC. The most widely used combination treatment is TACE combined with sorafenib (TACE-S) which has been confirmed to be better than monotherapy [9–11]. Besides the limited efficacy, sorafenib was also a huge economic burden because of the high price for many patients with advanced HCC in China. Thus, cheaper and effective alternatives to sorafenib are needed.

Apatinib is an antiangiogenic drug that targets the vascular endothelial growth factor receptor-2 (VEGFR-2) and exerts similar antitumor effects to sorafenib.

Apatinib can target VEGF-R-2, inhibit the activation of VEGFR-2, and then block its downstream signal by specifically competing for adenosine triphosphate binding site in the cell. It can also inhibit the proliferation migration and the tube formation of human umbilical vein endothelial cells, blocking the generation of aortic rings [12, 13]. In China, apatinib was approved for use in the treatment of gastric cancer and showed good efficacy [14, 15]. Recently, studies have shown that patients with unresectable HCC could get survival benefits from apatinib, especially apatinib combined with TACE (TACE-A) [16–18].

Research has shown that patients treated with TACE-S or TACE-A had better survival benefits than patients treated with monotherapy (TACE or sorafenib) [10, 19, 20]. However, there is still a lack of evidence about whether patients with TACE-S had similar survival benefits to patients treated with TACE-A. Thus, we conducted a study to compare the efficacy and safety of TACE-S and TACE-A in patients with advanced HCC.

## 2. Materials and Methods

### 2.1. Patient Selection

We retrospectively reviewed the medical records of 409 consecutive patients with advanced HCC who received TACE-S or TACE-A from June 2015 to December 2019 in our institution. Before the initial TACE procedure, TACE-S was firstly recommended. For patients who refused to receive sorafenib, TACE-A was recommended.

**Figure 1:**
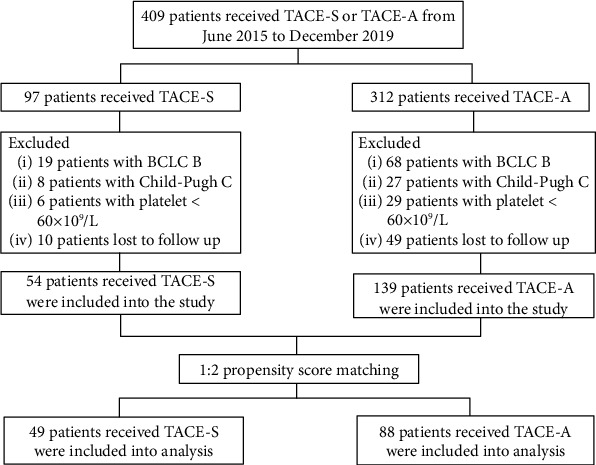
Flowchart of patient selection.

The inclusion criteria of the study were as follows: (1) patients were diagnosed as advanced HCC based on the EASL guideline; (2) the Child-Pugh score of patients was A or B; (3) the Eastern Cooperative Oncology Group of patients was 0, 1, or 2; and (4) the platelet counts of patients were higher than 60 × 10^9^/L. The exclusion criteria were as follows: (1) patients received TACE or sorafenib or apatinib before they were included in the study; (2) patients with tumors that were metastasized from other organs; and (3) patients with diffuse tumors which could not be evaluated (Figure 1). This study was conducted in accordance with the Declaration of Helsinki and approved by the institutional review board of the institution. Informed consent was waived by the board.

### 2.2. Treatments

#### 2.2.1. Transarterial Chemoembolization Procedure

TACE procedures were performed by three experienced radiologists with a minimum of 10 years of experience in interventional therapy. A 5 F catheter (Cook, Bloomington, IN, USA) was inserted into the hepatic artery. Then, a 3F micro-catheter (Progreat, Terumo, Tokyo, Japan) was used to insert the tumor-feeding arteries. Subsequently, lipiodol (Lipiodol Ultrafluido, Guerbet, Villepinte, France) was mixed with doxorubicin hydrochloride to create an emulsion at the ratio of 1 mL/2 mg. Depending on the size of the tumor and liver function, 5–20 mL emulsion was injected slowly into the tumor-feeding arteries until stasis of the feeding arteries of the tumor. If it was necessary, supplement embolization was performed using gelatin sponge particles (300–700 um, Cook, USA).

#### 2.2.2. Sorafenib and Apatinib Administration

In the TACE-S group, sorafenib was orally and continuously administered 400 mg twice daily when TACE was performed. In the TACE-A group, apatinib was orally taken 3–5 days after each TACE procedure at the starting dose of 500 mg/day. The dose adjustments were based on the tolerance to the drug. The grading of adverse events associated with apatinib and sorafenib was conducted according to the National Cancer Institute Common Terminology Criteria for Adverse Events (version 4.0). In the case of grade 3 or 4 apatinib- or sorafenib-related adverse events, the dose of apatinib was modified to 250 mg/day and the dose of sorafenib was adjusted to 400 mg once daily until the adverse effects were alleviated.

### 2.3. Endpoints

The primary endpoint of the study was overall survival (OS), which was defined as the time from the first TACE to patients' death or the end of the study. The secondary endpoints were progression-free survival (PFS) and disease control rate (DCR) 3 months after the initial TACE. The PFS was defined as the time from the initial TACE to the progression of the tumor, the time of patients' death, or the time of the end of the study. The DCR was defined as the proportion of patients with complete response (CR), partial response (PR), and stable response (SD) in patients with TACE-S or TACE-A based on the mRECIST criteria [21].

### 2.4. Follow-Up

All patients who were included in the study were followed up. In the study, patients were required to receive CT or MRI and laboratory tests every 4–6 weeks after the initial TACE for 6 months. After 6 months, patients were required to receive CT or MRI and laboratory tests every 2-3 months. The images of patients were reviewed by two radiologists (one with 24 years of radiographic experience and one with 20 years of radiographic experience) who were blinded to the treatment of the patients. If the results of their evaluation were different, another radiologist (with 31 years of radiographic experience) evaluated the results and gave the final results. When the CT or MRI image showed that the tumor remained or the tumor progressed, another TACE was recommended to these patients. The follow-up end time of the study was July 30, 2020.

### 2.5. Statistical Analysis

Continuous variables were compared by independent sample *t*-test or Mann–Whitney *U* test. Categorical variables were compared by the Chi-square test or Fisher's test between the two groups. Kaplan–Meier was used to plot the survival curves and the survival benefits in both groups and compared by log-rank. Adjusted Cox regression risk model was used to predict the potential factors which might influence the OS and PFS. Propensity score matching (PSM) was used to reduce selection bias. All characteristics were included in PSM analysis, and 1 : 2 ratio matching with an optimal caliper of 0.2 without replacement generated 139 patients (49 patients with TACE-S and 88 patients with TACE-A). The Landmark method was used in the study because it determines survival based on response status assessed at each specific time point. Because the Landmark is recommended in oncology guidelines for TACE-S/TACE-A analysis, a 12-month Landmark for OS and PFS was used in the study before and after PSM. All statistical analysis was conducted by SPSS 24.0 (IBM Corp, Armonk, NY, USA) and R 3.6.2.

## 3. Results

### 3.1. Characteristics of Patients

In this study, a total of 193 patients with advanced HCC who received TACE-S or TACE-A were included in the analysis. Among them, 54 patients received TACE-S and 139 patients received TACE-A. The median time of follow-up was 11.5 months (range, 2.5–58.1 months) in the TACE-S group and 11.5 months (range, 1–59.2 months) in the TACE-A group. In the TACE-S group, 43 patients of the 54 died and 120 patients of 139 patients died in the TACE-A group during the follow-up (Table 1).

### 3.2. Survival Outcomes and Tumor Response

**Figure 2:**
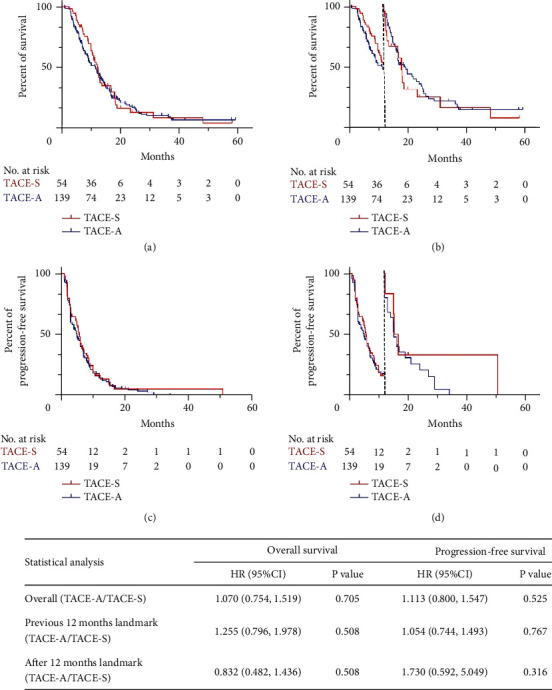
Kaplan–Meier curves and landmark analysis of OS and PFS before PSM. (a) Kaplan–Meier curve of OS. (b) Kaplan–Meier curve for landmark analysis of OS. (c) Kaplan–Meier curve of PFS. (d) Kaplan–Meier curve for landmark analysis of PFS. (Table) Univariable Cox regression analysis for OS and PFS.

**Figure 3:**
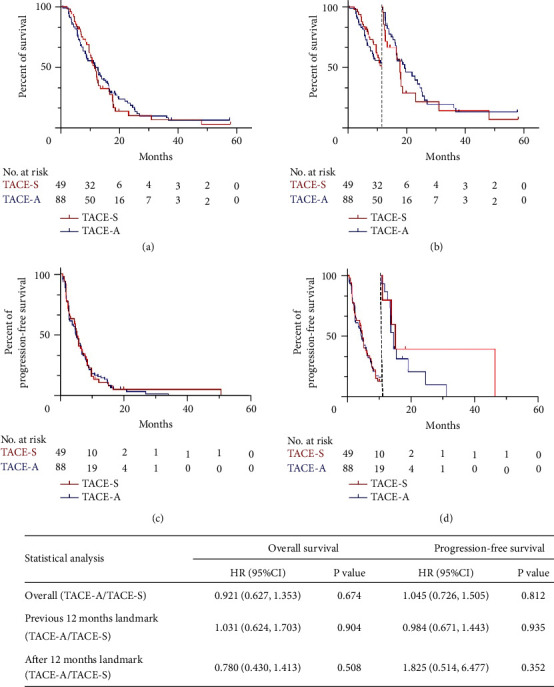
Kaplan–Meier curves and landmark analysis of OS and PFS after PSM. (a) Kaplan–Meier curve of OS.(b) Kaplan–Meier curve for landmark analysis of OS. (c) Kaplan–Meier curve of PFS. (d) Kaplan–Meier curve for landmark analysis of PFS. (Table) Univariable Cox regression analysis for OS and PFS.

Before PSM, the mOS was 12.4 months (95%CI: 10.7–14.1 months) in the TACE-S group and 11.5 months (95%CI: 8.8–14.2 months) in the TACE-A group (*P*=0.703). The mPFS was 5.7 months (95%CI: 5.0–6.4 months) in the TACE-S group and 5 months (95%CI: 3.8–6.2 months) in the TACE-A group (*P*=0.514) (Figure 2). The DCR in the previous 3 months was 56.8% (79/139) in the TACE-A group and 64.8% (35/54) in the TACE-S group (*P*=0.311). After PSM, the mOS and mPFS were 12.2 months (95%CI: 8.9–15.5 months) and 5.4 months (95%CI: 4.4–6.4 months) in the TACE-S group and 11.9 months (95%CI: 10.2–13.6 months), and 5.1 months (95%CI: 4.0–6.2 months) in the TACE-A group (*P*=0.672 and *P*=0.808) (Figure 3). The DCR was 60.2% (53/88) in the TACE-A and 63.3% (31/49) in the TACE-S group (*P*=0.726).

### 3.3. Landmark Analysis and Cox Regression Analysis

The Landmark analysis showed that TACE-A did not increase the mortality risk (HR: 1.255, 95%CI: 0.796–1.978, *P*=0.329) or tumor recurrence risk (HR: 1.054, 95%CI: 0.744–1.493, *P*=0.767) compared to TACE-S in the previous 12 months. After 12 months, TACE-A also did not increase the mortality risk (HR: 0.832, 95%CI: 0.482–1.436, *P*=0.508) or tumor recurrence risk (HR: 1.730, 95%CI: 0.592–5.049, *P*=0.316) compared to TACE-S (Figure 2). Similar results were presented after PSM. TACE-A did not increase the mortality risk (HR: 1.031, 95%CI: 0.624–1.703, *P*=0.904) or tumor recurrence risk (HR: 0.984, 95%CI: 0.671–1.443, *P*=0.935) compared to TACE-S in the previous 12 months. After 12 months, TACE-A also did not increase the mortality risk (HR: 0.780, 95%CI: 0.430–1.413, *P*=0.413) or tumor recurrence risk (HR: 1.825, 95%CI: 0.514–6.477, *P*=0.352) compared to TACE-S (Figure 3). In the Cox regression analysis, after adjustment for age, ALT, AST, hemoglobin, platelet, lymphocytes, neutrophils, tumor size, gender, HBV infection, AFP level, TACE session, tumor number, portal invasion, extrahepatic metastases, cirrhosis, Child-Pugh score, and Eastern Cooperative Oncology (ECOG), TACE-A did not increase the mortality risk (HR: 0.908, 95%CI: 0.620–1.330, *P*=0.620) or tumor recurrence risk (HR: 0.906, 95%CI: 0.634–1.295, *P*=0.589) compared to TACE-S before PSM (Table 2). In the Landmark analysis for 12 months, TACE-A did not increase the mortality risk (HR: 1.086, 95%CI: 0.663–1.780, *P*=0.744) or tumor recurrence risk (HR: 0.906, 95%CI: 0.624–1.314, *P*=0.601) compared to TACE-S. After 12 months, TACE-A did not increase the mortality risk (HR: 0.627, 95%CI: 0.307–1.281, *P*=0.200) but reduced the tumor recurrence risk (HR: 0.044, 95%CI: 0.002–0.810, *P*=0.036) compared to TACE-S (Table 2).

### 3.4. Subgroup Analysis

**Figure 4:**
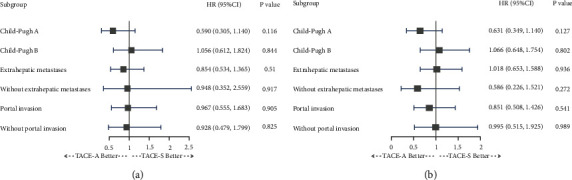
Adjusted Cox regression analysis for subgroups analysis before PSM, adjusted for age, ALT, AST, HB, PLT, LYM, NEU, size, gender, HBV, APF, TACE session, tumor number, cirrhosis, and ECOG. (a) Subgroups analysis for OS. (b) Subgroup analysis for PFS.

In the adjusted Cox regression analysis, after adjustment for age, ALT, AST, hemoglobin, platelet, lymphocytes, neutrophils, tumor size, gender, HBV infection, AFP level, TACE session, tumor number, cirrhosis, and ECOG, TACE-A did not increase the mortality risk (Child-Pugh A: HR: 0.590, 95%CI: 0.305–1.140, *P*=0.116; Child-Pugh B: HR: 1.056, 95%CI: 0.612–1.824, *P*=0.844) or tumor recurrence risk (Child-Pugh A: HR: 0.631, 95%CI: 0.349–1.140, *P*=0.127; Child-Pugh B: HR: 1.066, 95%CI: 0.648–1.754, *P*=0.802) compared to TACE-S in the patients with Child-Pugh A and B scores. Similarly, TACE-A did not increase the mortality risk (with extrahepatic metastases: HR: 0.854, 95%CI: 0.534–1.365, *P*=0.510; without extrahepatic metastases: HR: 0.948, 95%CI: 0.352–2.559, *P*=0.917) or tumor recurrence risk (with extrahepatic metastases: HR: 1.018, 95%CI: 0.653–1.588, *P*=0.936; without extrahepatic metastases: HR: 0.586, 95%CI: 0.226–1.521, *P*=0.272) compared to TACE-S in the patients with extrahepatic metastases and without extrahepatic metastases. Again, TACE-A also did not increase mortality risk (with portal invasion: HR: 0.967, 95%CI: 0.555–1.683, *P*=0.905; without portal invasion: HR: 0.928, 95%CI: 0.479–1.799, *P*=0.825) or tumor recurrence risk (with portal invasion: HR: 0.851, 95%CI: 0.508–1.426, *P*=0.541; without portal invasion: HR: 0.995, 95%CI: 0.515–1.925, *P*=0.989) compared with TACE-S in the patients with portal invasion and without portal invasion before PSM (Figure 4).

### 3.5. Safety of Patients with TACE-S or TACE-S

In the study, the adverse events of patients with TACE and sorafenib and apatinib were evaluated. For all grades of adverse events, there was no statistically significant difference of fever (*P*=0.216), abdominal pain (*P*=0.886), nausea (*P*=0.443), vomiting (*P*=0.235), anorexia (*P*=0.692), diarrhea (*P*=0.749), hypertension (*P*=0.085), fatigue (*P*=0.343), hand-foot reaction (*P*=0.715), gastrointestinal hemorrhage (*P* > 0.999), headache (*P*=0.921), and proteinuria (*P*=0.892) in the TACE-S group and TACE-A group. Similarly, there was no statistically significant difference of relative adverse events (grades ≥3) between the two groups (all *P* > 0.05) (Table 3).

## 4. Discussion

Although the SHARP and ORIENTAL clinical trials showed that patients with advanced HCC who received sorafenib had better survival benefits than a placebo group, the response rate of patients who received sorafenib was still low [8, 22–24]. Thus, TACE combined with sorafenib was used in the treatment of advanced HCC more widely and showed good efficacy. However, due to the low response rate and high cost of sorafenib, another selective drug, apatinib, was used in the treatment of advanced HCC. Several studies have showed that patients treated with TACE-A had improved survival compared to patients with single treatment. However, any differences in efficacy between the patients with TACE-S and TACE-A were still unclear. Thus, the aim of the study was to compare the efficacy and the safety of advanced HCC patients treated with either TACE-S or TACE-A.

Previous studies have shown that the mOS of advanced HCC patients with TACE-S ranged from 11.2 months to 17.5 months, and the median time to progression (mTTP) or mPFS ranged from 4.3 months to 7.6 months [19, 20, 25–28]. The mOS of advanced HCC patients with TACE-A ranged from 12 to 22 months and mTTP from 6.1 to 9.5 months [18, 29, 30]. In the current study, we got similar results. The mOS and mPFS of patients with TACE-S were 12.4 months and 5.7 months, respectively. Compared with previous studies analyzing the survival of patients who received TACE plus sorafenib, the mOS and mPFS of the current study were shorter than the mOS (16.5 months and 17.5 months) and mPFS (7 months) or mTTP (7 months) in the two studies [19, 20]. The reason might be the heterogeneity of the included patients. The current study included 57.4% of patients with Child-Pugh B and 72.2% of patients with extrahepatic metastases. However, the study conducted by Koch et al. included 26% of patients with Child-Pugh B and 41% of patients with extrahepatic spread. The study conducted by Kim et al. included 8% of patients with Child-Pugh B. The mOS and mPFS of patients with TACE-A were 11.5 months and 5 months, respectively. Before and after PSM, there was no statistically significant difference of mOS and mPFS between the patients with TACE-S and the patients with TACE-A. Some patients with HCC who received TACE could realize complete embolization, which increases the hypoxia of tumor cells. The hypoxic microenvironment of tumor cells can make tumor cells generate proangiogenic factors, which leads to tumor angiogenesis and tumor progression. Apatinib is an antiangiogenic drug. It can prevent angiogenesis after patients receive TACE and prevent further tumor progression, which might be the reason why advanced HCC patients who received TACE-A had similar survival benefits to patients who received TACE-S.

In the study, the Landmark was used to mitigate guarantee-time bias, and a 12-month period was used [31, 32]. The study results showed that patients with TACE-A did not have an increased mortality risk or tumor recurrence compared with patients treated with TACE-S in the univariable regression and multivariable regression analysis at the previous 12 months and 12 months after PSM. The results indicate that the tumor response of patients who received TACE-A was similar to patients who received TACE-S at both early and late times after the initial TACE. In the adjusted Cox regression analysis for all patients, all factors were included in the analysis to reduce potential factors which might influence the overall survival and tumor progression. After reducing the potential influencing factors, TACE-A did not increase mortality risk or tumor recurrence relative to TACE-S. Previous studies showed that the liver function, tumor portal invasion, and distant metastases might influence the survival of patients with HCC. Thus, in the current study, the subgroups analysis divided patients into six groups, such as patients with Child-Pugh A score, patients with Child-Pugh B score, patients with portal invasion, patients without portal invasion, patients with tumor distant metastases, and patients without distant metastases. The adjusted Cox regression analysis showed that TACE-A did not increase mortality risk or tumor recurrence risk compared with TACE-S in different groups. The results of subgroups analysis indicated that the different statuses of patients with advanced HCC who received TACE-A could still get similar survival benefits compared with patients who received TACE-S.

In the study, the adverse events related to TACE or apatinib or sorafenib were evaluated. The III and IV grades of adverse events in the TACE-A treatment group were not higher than the TACE-S treatment group. And after reducing the dose administration of apatinib, the adverse events of patients with TACE-A and TACE-S were relieved. The results of this study showed that apatinib is a well-tolerated treatment option with an acceptable safety profile for patients with advanced HCC compared with sorafenib.

Some limitations existed in the study. Firstly, the study was a retrospective study, which led to the existence of selection bias. However, the PSM was used in the study to reduce selection bias. Secondly, the number of patients enrolled in the study was small, especially the sample of patients with TACE-S, which might influence the accuracy of the conclusion. Finally, the selection of drugs might be influenced by the patients' willingness, which might result in some patients receiving suboptimal treatments. However, until now, there have been no criteria indicating which kind of advanced HCC patients are suitable for sorafenib or apatinib. Consequently, more studies are needed to confirm the results of the study.

## 5. Conclusion

The study showed that patients with advanced HCC who received TACE-A had comparable survival benefits to patients who received TACE-S. TACE-A might be a good choice for patients who were unwilling to receive TACE-S.

## Figures and Tables

**Table 1:** Baseline characteristics of patients with TACE-S and TACE-A before PSM and after PSM.

Characteristics	Before matching			After matching		
	TACE-S (mean ± SD) (*N* = 54/%)	TACE-A (mean ± SD) (*N* = 139/%)	*P* value	TACE-S (mean ± SD) (*N* = 49/%)	TACE-A (mean ± SD) (*N* = 88/%)	*P* value
Age	53.1 ± 10.6	51.2 ± 9.8	0.243	52.8 ± 10.7	51.8 ± 8.8	0.593
ALT	57.5 ± 78.3	60.3 ± 69.0	0.808	57.9 ± 82.0	51.2 ± 57.5	0.579
AST	73.8 ± 87.5	78.2 ± 147.1	0.839	76.6 ± 91.5	52.9 ± 45.4	0.194a
Hemoglobin	128.2 ± 21.6	126.4 ± 20.2	0.588	127.7 ± 22.5	125.2 ± 21.2	0.515
Platelet	155.6 ± 68.7	164.5 ± 80.7	0.475	157.0 ± 71.4	163.0 ± 78.2	0.660
Lymphocytes	1.3 ± 0.7	1.2 ± 0.5	0.396	1.2 ± 0.6	1.2 ± 0.6	0.695
Neutrophils	3.4 ± 1.9	3.7 ± 1.9	0.363	3.6 ± 1.9	3.6 ± 1.8	0.927
Tumor size	8.2 ± 4.4	9.0 ± 4.3	0.278	8.5 ± 4.5	8.1 ± 4.0	0.589
Gender						
Male	51 (94.4)	119 (85.6)	0.089	46 (93.9)	80 (90.9)	0.776
Female	3 (5.6)	20 (14.4)		3 (6.1)	8 (9.1)	
HBV infection						
Yes	41 (75.9)	108 (77.7)	0.792	39 (79.6)	63 (71.6)	0.303
No	13 (24.1)	31 (22.3)		10 (20.4)	25 (28.4)	
AFP						
<200	30 (55.6)	55 (39.6)	0.045	25 (51)	42 (47.7)	0.712
≥200	24 (44.4)	84 (60.4)		24 (49)	46 (52.3)	
TACE session						
1	11 (20.4)	24 (17.3)	0.615	10 (20.4)	14 (15.9)	0.507
≥2	43 (79.6)	115 (82.7)		39 (79.6)	74 (84.1)	
Tumor number						
1	15 (27.8)	43 (30.9)	0.688	14 (28.6)	24 (27.3)	0.871
≥2	39 (72.2)	96 (69.1)		35 (71.4)	64 (72.7)	
Portal invasion						
Yes	31 (57.4)	84 (60.4)	0.701	28 (57.1)	46 (52.3)	0.584
No	23 (42.6)	55 (39.6)		21 (42.9)	42 (47.7)	
Extrahepatic metastasis						
Yes	39 (72.2)	100 (71.9)	0.969	36 (73.5)	67 (76.1)	0.729
No	15 (27.8)	39 (28.1)		13 (26.5)	21 (23.9)	
Cirrhosis						
Yes	34 (63)	85 (61.2)	0.816	31 (63.3)	53 (60.2)	0.726
No	20 (37)	54 (38.8)		18 (27.7)	35 (39.8)	
Child-Pugh						
A	23 (42.6)	66 (47.5)	0.541	21 (42.9)	42 (47.7)	0.584
B	31 (57,4)	73 (52.5)		28 (57.1)	46 (52.3)	
ECOG						
0	18 (33.3)	22 (15.8)	0.021	14 (28.6)	21 (23.9)	0.675
1	29 (53.7)	87 (62.6)		28 (57.1)	57 (64.8)	
2	7 (13)	30 (21.6)		7 (14,3)	10 (11.3)	

Abbreviations: TACE-S: transarterial chemoembolization combined with sorafenib; TACE-A: transarterial chemoembolization combined with apatinib; ALT: alanine aminotransferase; AST: aspartate aminotransferase; AFP: alpha-fetoprotein; ECOG: Eastern Cooperative Oncology Group.

**Table 2:** Adjusted Cox regression for OS and PFS before PSM, adjusted for age, ALT, AST, hemoglobin, platelet, lymphocytes, neutrophils, tumor size, gender, HBV infection, AFP level, TACE session, tumor number, portal invasion, extrahepatic metastases, cirrhosis, Child-Pugh, and ECOG.

Characteristics	Overall survival		Progression-free survival	
	HR (95%CI)	*P* value	HR (95%CI)	*P* value
Overall				
TACE-S	Reference	0.620	Reference	0.589
TACE-A	0.908 (0.620,1.330)		0.906 (0.634,1.295)	

Before 12-month Landmark				
TACE-S	Reference	0.744	Reference	0.601
TACE-A	1.086 (0.663,1.780)		0.906 (0.624,1.314)	

Before 12-month Landmark				
TACE-S	Reference	0.200	Reference	0.036
TACE-A	0.627 (0.307,1.281)		0.044 (0.002,0.810)	

Abbreviations: TACE-S: transarterial chemoembolization combined with sorafenib; TACE-A: transarterial chemoembolization combined with apatinib.

**Table 3:** Adverse events of patients after receiving TACE-S and TACE-A before PSM.

Adverse events	All grades			Grades ≥3		
	TACE-S	TACE-A	*P* value	TACE-S	TACE-A	*P* value
Fever	23 (42.6)	73 (52.5)	0.216	1 (1.9)	2 (3.7)	>0.999
Abdominal pain	44 (81.5)	112 (80.6)	0.886	2 (3.7)	5 (3.6)	0.972
Nausea	35 (64.8)	98 (70.5)	0.443	1 (1.9)	4 (2.9)	0.677
Vomiting	24 (44.4)	75 (54)	0.235	1 (1.9)	3 (2.2)	0.892
Anorexia	22 (40.7)	61 (46.1)	0.692	0 (0)	0 (0)	>0.999
Diarrhea	12 (22.2)	28 (20.1)	0.749	0 (0)	0 (0)	>0.999
Hypertension	19 (35.2)	68 (48.9)	0.085	1 (1.9)	2 (1.4)	>0.999
Fatigue	6 (11.1)	23 (16.5)	0.343	0 (0)	0 (0)	>0.999
Hand-foot skin reaction	7 (13.0)	21 (15.1)	0.704	2 (3.7)	5 (3.6)	0.687
Gastrointestinal hemorrhage	0 (0)	2 (1.4)	>0.999	0 (0)	0 (0)	>0.999
Headache	9 (16.7)	24 (17.3)	0.921	0 (0)	1 (0.7)	>0.999
Proteinuria	1 (1.9)	3 (2.2)	0.892	0 (0)	0 (0)	>0.999

Abbreviations: TACE-S: transarterial chemoembolization combined with sorafenib; TACE-A: transarterial chemoembolization combined with apatinib.

## Data Availability

The data used in the study are available from the corresponding authors on reasonable request.

## Abbreviations

TACE-S: Transarterial chemoembolization combined with sorafenib
TACE-A: Transarterial chemoembolization combined with apatinib
HCC: Hepatocellular carcinoma
BCLC: Barcelona clinic liver cancer
OS: Overall survival
PFS: Progression-free survival
DCR: Disease control rate
PSM: Propensity score matching
ECOG: Eastern cooperative oncology group
AFP: Alpha-fetoprotein.
